# MIS-TLIF for a gas-containing discal cyst with adjacent -lumbar spondylolisthesis: a case report and literature review

**DOI:** 10.3389/fsurg.2025.1626636

**Published:** 2025-08-26

**Authors:** Haoyun Huang, Guangye Li, Junwen Deng, Rigao Chen, Yi Zhou

**Affiliations:** ^1^ School of Clinical Medicine, Chengdu University of Traditional Chinese Medicine, Chengdu, China; ^2^ Department of Orthopedics, Hospital of Chengdu University of Traditional Chinese Medicine, Chengdu, China

**Keywords:** MIS-TLIF, discal cyst, gas-containing, lumbar spondylolisthesis, adjacent segment, case report

## Abstract

**Introduction:**

Gas-containing lumbar disc cysts are a rare cause of neurogenic pain. These cysts typically occur in middle-aged and elderly patients and are predominantly associated with lumbar disc degeneration, presenting with sensory and motor deficits in the affected dermatomes. The prevalence remains unclear due to the limited number of reported cases. Instances involving concomitant lumbar spondylolisthesis at adjacent segments are extremely rare and poorly documented in the current literature.

**Case presentation:**

The 67-year-old woman presenting with recurrent lower back pain, numbness, and swelling in the left lower limb for over 6 months. Previous conservative treatments yielded minimal relief. DR imaging showed anterior displacement of the L4 vertebral body and reduced L5/S1 intervertebral height. CT imaging confirmed L4 anterior slippage with L4/5 segmental spinal stenosis, a disc vacuum phenomenon at L5/S1, and a gas-containing cyst at the left posterior margin of the intervertebral disc. MRI imaging revealed a low-signal area on T1-weighted imaging/T2-weighted imaging sequences at the cyst location. A preliminary diagnosis of L5/S1 gas-containing disc cyst with adjacent L4/5 spondylolisthesis was established based on comprehensive clinical assessments. And a two-segment minimally invasive transforaminal lumbar interbody fusion was performed at L4/5 and L5/S1.

**Result:**

The patient experienced significant symptom relief, with postoperative CT confirming cyst resolution, restored L5/S1 intervertebral height, and L4/5 stability. At 1-month follow-up, the patient reported minimal pain. By 3 months, lower extremity symptoms had fully resolved.

**Conclusion:**

These findings suggest that fusion surgery might be a superior treatment option for cases involving discal cysts with spondylolisthesis, particularly when clinical evidence of vertebral instability is present.

## 1 Introduction

Gas-containing lumbar disc cysts are a rare cause of lumbar radiculopathy with distinctive epidemiologic features, predominantly observed in Asian male populations (1). Their pathogenesis is closely linked to damage and degenerative changes in the annulus fibrosus of the intervertebral discs (2). Typical clinical manifestations include signs of nerve compression, such as radiating pain, sensory abnormalities, and neurogenic intermittent claudication in the affected dermatomes (3). Surgery is generally effective, with cystectomy being the most frequently performed procedure. However, due to the rarity of this condition, there is no established consensus on the optimal surgical approach (4). There is a limited number of case reports on this phenomenon, most of which describe simple cysts. The co-occurrence of a gas-containing disc cyst with adjacent-segment degenerative lumbar spondylolisthesis is particularly uncommon and insufficiently documented. This study presents the case of an L5/S1 gas-containing disc cyst combined with L4/5 spondylolisthesis, successfully managed with minimally invasive transforaminal lumbar interbody fusion (MIS-TLIF). Through this case and a review of the literature, the clinical decision-making process in managing such complex cases was examined.

## 2 Case presentation

**Figure 1:**
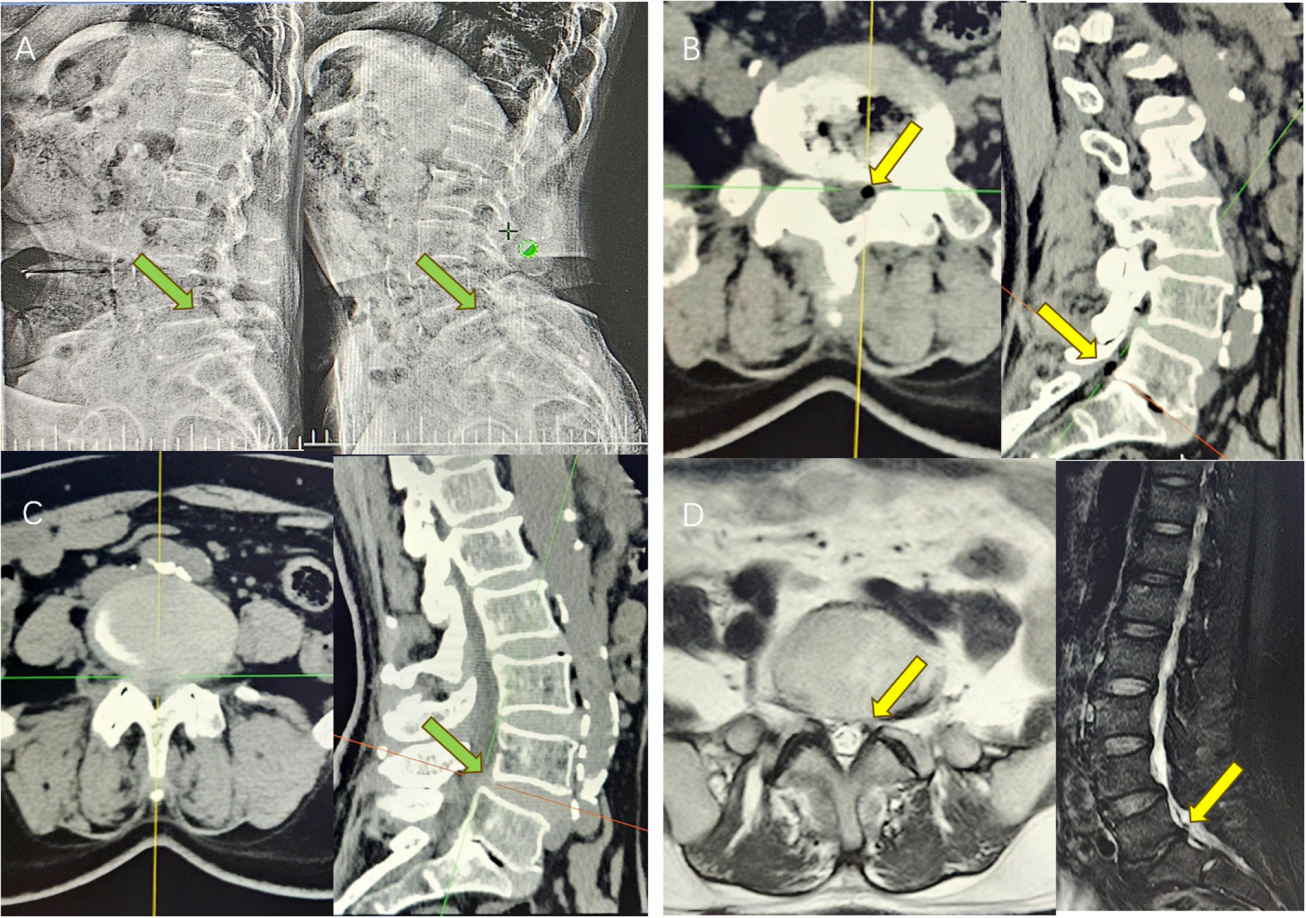
**(A)** Preoperative dynamic DR showed that the intervertebral height of L5/S1 segment was reduced, and L4 slipped forward I° (green arrow); **(B)** preoperative CT suggested that the left side of L5/S1 contained a pneumatic cyst, with the corresponding nerve root compression (yellow arrow). The intervertebral height of the corresponding segment was reduced, with the sign of intervertebral disc vacuum; **(C)** Preoperative CT showed that the L4 slipped forward I°, with spinal stenosis at the corresponding level (green arrow); **(D)** Preoperative MRI showed hypointense T1/T2 images on the left side of L5/S1 (yellow arrow).

**Figure 2:**
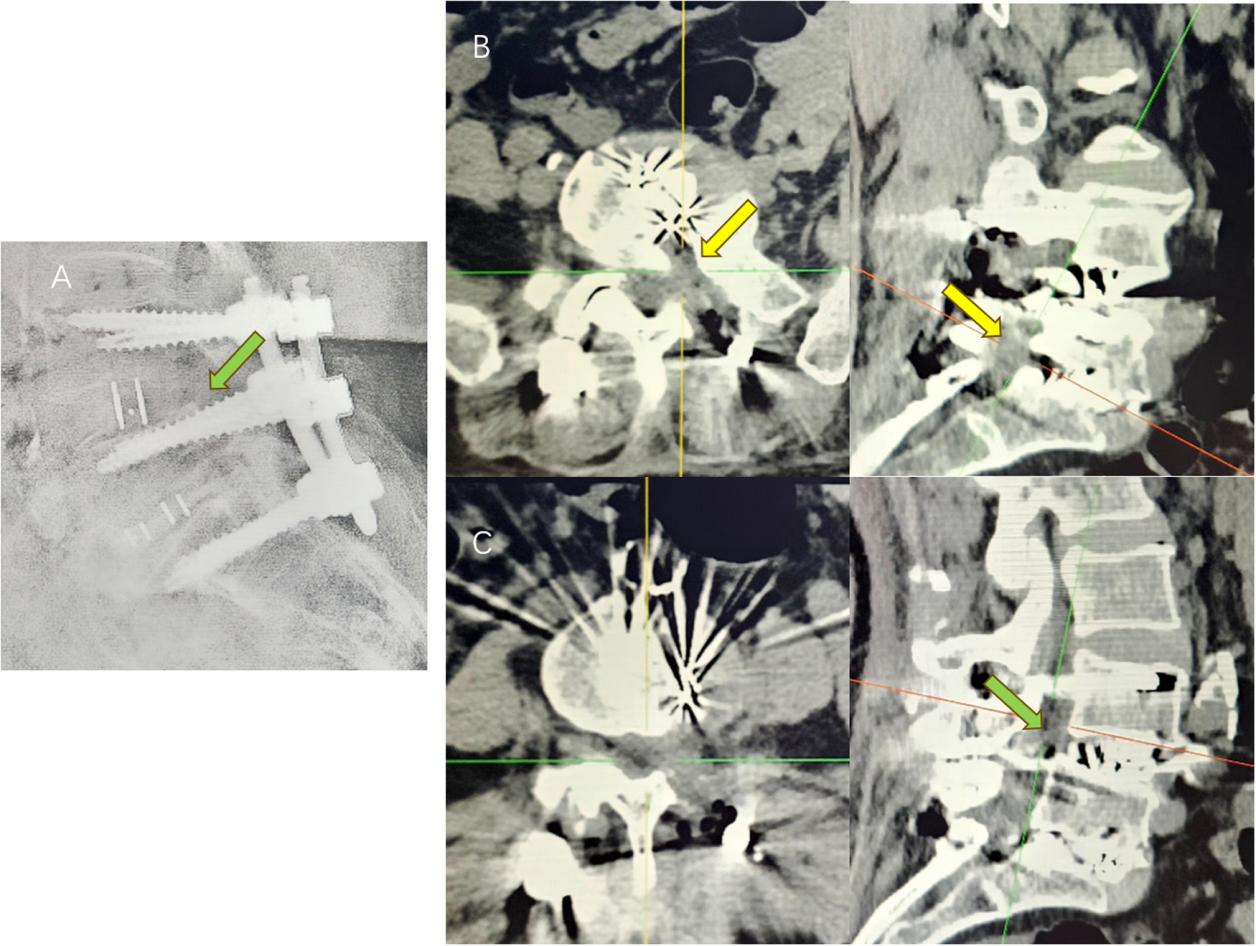
**(A)** postoperative DR showed that the intervertebral height of the L5/S1 segment was restored and the L4 slip was corrected; **(B)** postoperative CT showed that the cyst on the left side of the L5/S1 had disappeared (yellow arrow), and the intervertebral height of the corresponding segment was restored; **(C)** postoperative CT showed that the L4 slip was corrected (green arrow).

The patient was a 67-year-old woman presenting with recurrent lower back pain, numbness, and swelling in the left lower limb for over 6 months [visual analogue scale (VAS) score = 7]. The pain was radiating in nature, extending to the posterior and lateral aspects of the left calf, with intermittent radiating pain in the lateral aspect of the right calf. Symptoms worsened with ambulation, limiting walking distance to approximately 50 meters due to intermittent claudication. Previous conservative treatments, including physical therapy, yielded minimal relief. The patient had no history of lumbar trauma or surgery. Neurological examination revealed a strongly positive Lasègue's sign on the left and a positive response on the right. Radiographic imaging (Figure 1A) showed anterior displacement of the L4 vertebral body and reduced L5/S1 intervertebral height, leading to foraminal stenosis. Computed tomography (CT) imaging confirmed L4 anterior slippage with L4/5 segmental spinal stenosis (Figure 1B), a disc vacuum phenomenon at L5/S1, and a gas-containing cyst at the left posterior margin of the intervertebral disc (Figure 1C). Magnetic resonance imaging revealed a low-signal area on T1-weighted imaging/T2-weighted imaging sequences at the cyst location, consistent with a gas-containing discal cyst (Figure 1D). Clinically, the patient exhibited low back pain with posterolateral radiation to the left lower limb and lateral radiation to the right, consistent with L4 and L5 nerve root involvement. Based on neurological and imaging findings, the symptoms were attributed to compression from the L5/S1 pneumatic cyst and spinal stenosis secondary to L4/5 spondylolisthesis. A two-segment MIS-TLIF was performed at L4/5 and L5/S1 (preoperative VAS score = 7). During surgery, the degenerative disc tissue and cyst wall were excised (Figure 2B), the slipped vertebrae were realigned (Figure 2C), and interbody fusion, along with placement of pedicle screws, was performed to restore spinal stability (Figure 2A). Postoperatively, the patient experienced significant symptom relief (postoperative VAS score = 2), with CT confirming cyst resolution, restored L5/S1 intervertebral height, and L4/5 stability (Figure 2). At 1-month follow-up, the patient reported minimal pain (VAS = 1). By 3 months, intermittent lumbar soreness and distension persisted; however, lower extremity symptoms had fully resolved.

## 3 Discussion

Reports of gas-containing disc cysts in the literature are exceedingly rare, with cases involving concurrent lumbar spondylolisthesis being even less common. The aetiology and pathogenesis of these lesions remain incompletely understood. It is generally accepted that such cysts represent a rare complication of the disc vacuum phenomenon and are strongly associated with disc degeneration (5). Kakitsubata and Kanna et al. proposed a widely referenced pathological hypothesis suggesting that cyclic changes in intradiscal pressure, driven by spinal activity, contribute to cyst formation in degenerated discs. According to this model, annular fissures develop a “unidirectional flap” due to the structural properties of the local fibrous tissue. During spinal flexion and weight-bearing activities, increased intradiscal pressure causes the fissure to close (valve closes), while extension and pressure reduction lead to fissure opening (valve opens) (6). This cyclical process facilitates the movement of intradiscal gas through a pumping-like effect. When gas migrates toward a weakened area of the annulus fibrosus, it might protrude into the spinal canal, forming a cyst (7). The development of adjacent segment spondylolisthesis might result from biomechanical compensation following loss of intervertebral disc height. Disc vacuum signs typically accompany advanced degeneration, including nucleus pulposus dehydration and annular fissuring, leading to reduced disc height and compromised load-bearing capacity, thereby limiting mobility in the segment (8). This restriction in mobility transfers mechanical stresses to adjacent segments, which compensate through increased mobility. Over time, this compensatory hypermobility accelerates degeneration in the adjacent segments, creating a cascading response of mechanical imbalance e (8).

While conservative management is typically employed for patients with mild symptoms, surgery remains the gold standard for severe cases unresponsive to conservative treatment (9). However, the optimal indications and choice of surgical approach remain controversial. As summarized in Table 1, current literature supports microscopic or spinal endoscopic cystectomy as an effective and widely accepted option for patients with symptomatic cysts refractory to conservative therapy. Wang et al. retrospectively analysed nine cases of microscopic cystectomy and observed that the clinical presentation closely resembled that of lumbar disc herniation. This finding suggests that surgical indications for cystectomy might parallel those for herniated disc surgery (10). Park and Suo et al. also reported successful outcomes with microscopic and spinal endoscopic cystectomy (11, 12), emphasising the simplicity, minimal invasiveness, and low recurrence rate of the procedure. CT-guided percutaneous aspiration represents another treatment modality. Kang et al. employed CT-guided percutaneous transluminal aspiration in eight patients, achieving significant short-term symptomatic relief (13). However, one patient experienced recurrence and subsequently required open surgical intervention. It is noteworthy that the existing literature almost exclusively addresses cases involving simple cystic compression. Complex cases associated with spinal instability remain largely undocumented and lack systematic evaluation. The distinguishing characteristic of this case is the coexistence of an L5/S1 gas-containing cyst with L4/5 spondylolisthesis, contributing to the patient's symptoms. Given that cystectomy alone was insufficient for symptom relief, a bi-segmental fusion was performed. In cases where symptoms are solely due to simple cystic compression, isolated cystectomy can yield satisfactory outcomes. However, when cysts are accompanied by lumbar instability, careful evaluation is essential to determine the primary source of symptoms. If instability is identified as a contributing factor, cystectomy combined with lumbar interbody fusion might offer a more effective solution. MIS-TLIF not only facilitates complete removal but also restores spinal stability and biomechanical balance. Although more invasive than cystectomy alone, MIS-TLIF has been associated with more comprehensive symptom relief and improved long-term outcomes. Compared with open fusion, MIS-TLIF offers several advantages, including reduced trauma, less intraoperative blood loss, reduced postoperative pain, a shorter operative time, faster recovery, and a lower reoperation rate (14). Based on these findings, fusion surgery may represent a more suitable therapeutic strategy for complex cases involving cyst formation and spinal instability.

**Table 1:** Summary of the reported cases.

Article	Article type	Cases number	Level	Symptom	Cysts type	Lumbar stability	Surgical strategy
Perillo et al. (15)	Case report	1	L3/4	Low back pain and radiating leg pain	Gas	Stable	Micro-discectomy and cystectomy
Hu et al. (16)	Case report	1	L5/S1	Low back pain and radiating leg pain	Gas	Stable	PELD
Zhu and He (17)	Case report	3	L4/5	Low back pain and radiating leg pain	Gas	Stable	TPED
Mathon et al. (18)	Case report	1	L4/5	Low back pain and radiating leg pain	Gas	Stable	TLIF
Arslan et al. (19)	Case report	1	L3/4	Low back pain and radiating leg pain	Gas	Stable	Micro- cystectomy
Wang et al. (10)	Retrospective study	9	L2/3 L3/4 L4/5 L5/S1	Low back pain and radiating leg pain	Gas	Stable	Micro- cystectomy
Yun et al. (20)	Case report	2	L4/5 L5/S1	Low back pain and radiating leg pain	Gas	Stable	Micro- cystectomy
Lee et al. (21)	Case report	2	L4/5 L5/S1	Low back pain and radiating leg pain	Gas	Stable	Micro- cystectomy

## 4 Conclusion

This study is the first to report a two-segment MIS-TLIF for treating a lumbar intervertebral disc cyst combined with adjacent segmental spondylolisthesis, yielding favorable clinical outcomes. These findings suggest that fusion surgery might be a superior treatment option for cases involving discal cysts with spondylolisthesis, particularly when clinical evidence of vertebral instability is present.

## Data Availability

The original contributions presented in the study are included in the article/Supplementary Material, further inquiries can be directed to the corresponding authors.
